# Ferrocene Molecular Architectures Grafted on Si(111): A Theoretical Calculation of the Standard Oxidation Potentials and Electron Transfer Rate Constant

**DOI:** 10.3390/ma10101109

**Published:** 2017-09-21

**Authors:** Claudio Fontanesi, Massimo Innocenti, Davide Vanossi, Enrico Da Como

**Affiliations:** 1 Department of Engineering “Enzo Ferrari”, DIEF, University of Modena and Reggio Emilia, via Vivarelli 10, 41125 Modena, Italy; 2 Department of Chemistry, University of Firenze, via della Lastruccia 3, 50019 Sesto Fiorentino, Italy; m.innocenti@unifi.it; 3 Department of Chemical and Geological Sciences, DSCG, University of Modena and Reggio Emilia, via Campi 103, 41125 Modena, Italy; davide.vanossi@unimore.it; 4 Department of Physics, University of Bath, Claverton Down, Bath BA2 7AY, UK

**Keywords:** CDFT, electron transfer, ferrocene, Marcus theory

## Abstract

The standard oxidation potential and the electron transfer (ET) rate constants of two silicon-based hybrid interfaces, Si(111)/organic-spacer/Ferrocene, are theoretically calculated and assessed. The dynamics of the electrochemical driven ET process is modeled in terms of the classical donor/acceptor scheme within the framework of “Marcus theory”. The ET rate constants, $k_{ET}$, are determined following calculation of the electron transfer matrix element, $V_{RP}$, together with the knowledge of the energy of the neutral and charge separated systems. The recently introduced Constrained Density Functional Theory (CDFT) method is exploited to optimize the structure and determine the energy of the charge separated species. Calculated ET rate constants are $k_{ET}=77.8\;s^{-1}$ and $k_{ET}=1.3\times 10^{-9}\;s^{-1}$, in the case of the short and long organic-spacer, respectively.

## 1. Introduction

**Scheme 1:**
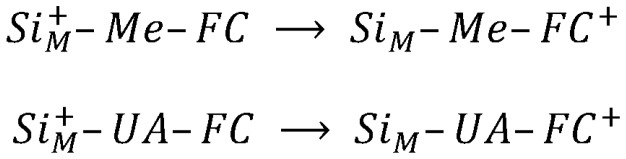
Charge localization in the two-diabatic-states mechanism.

Beyond any possible doubt, Silicon is a fundamental material of utmost importance for both applicative purposes and pure science [1]. In this context, recent research activity shows a growing interest in the area of hybrid, silicon-based, molecular electronics, which is driven by possible technological applications such as biosensors, photovoltaic cells, and optoelectronic devices [2,3,4,5,6]. Different methodologies exist for the preparation of hybrid silicon-based interfaces, relying on the covalent grafting of organic molecules, and these are generally based on ultra-high vacuum (UHV) depositions [7,8,9], wet chemistry exploiting UV curing [10,11], electrochemical-based methodologies [12,13]. Remarkably, the study of the electron transfer (ET) process for ferrocene derivatives grafted on silicon allows for a comparison with results obtained for similar systems self-assembled on gold [14]. Within this picture, the ferrocene/silicon interface is an intriguing “model” system representative of the classical Donor-Spacer-Acceptor molecular system. This is due to ferrocene’s peculiar properties: low oxidation potential, almost ideal ET reversibility, fast ET rate, and only two stable redox states [15]. In particular, chemi-adsorbed ferrocene moieties on silicon surfaces hold the promise to be exploited as a memory elements, where the ferrocene redox center is used as the charge storage component and the oxidation states (neutral or oxidized) as the two bits [16]. Similar molecular systems were experimentally characterized in a very extensive way, as far as the ET process is concerned [17,18,19,20,21,22]. However, from a theoretical point of view, the fundamental knowledge of the ET dynamics, at a hybrid interface, is a subject still open to discussion [23,24]. In this paper, we study two different ferrocene molecular architectures grafted via a covalent bond onto a Si(111) surface. The two systems here studied are characterized by the different length of spacer (an alkyl-chain) linking the ferrocene moiety to the silicon: (1) methanol-ferrocene directly grafted on the surface (i.e., –O–CH_2_– short spacer) and (2) ferrocene grafted through a 10 CH_2_ methylene group (UA) moiety (i.e., –(CH_2_)_10_–COO–CH_2_– long spacer). The ET process is considered to occur from the ferrocene-donor (D) to the silicon-acceptor (A), as it is represented in a two-diabatic-states reaction mechanism ($D–A\;\to \;D^{+}–A^{-}$), Scheme 1 [23,25]:

where *Me* and *UA* stand for –O–CH_2_– and –(CH_2_)_10_–COO–CH_2_– moieties (spacers), respectively; and *Si_M_* stands for the silicon surface. Note that the two hybrid interfaces devised in this paper can be easily produced in most laboratories: $Si_{M}–Me–FC$ can be prepared by heating the Si(111) hydrogen terminated surface in direct contact with methanol ferrocene, while $Si_{M}–UA–FC$ can be obtained following a two-step procedure. The first step is the electrochemical grafting of 11-Bromoundecanoic acid; the second step is the condensation reaction of the methanol ferrocene with the dangling carboxylic group [26,27,28,29]. The scope of this work is the calculation of the ET rate constant, to be compared with experimental electrochemical results. This goal is pursued by exploiting the Marcus theory [30,31], taking advantage of theoretical results calculated by using the Constrained Density Functional Theory (CDFT) calculations. CDFT calculations allow us to evaluate the energy and electronic structure of molecular systems featuring a discrete net charge localization on suitably selected moieties [32,33]. Theoretical calculations are then discussed in the view of the available experimental results [10,12,19,34,35,36,37,38,39]. It is important to note that the theoretical calculation of ET rate constants are rarely reported in the literature [40,41]. Moreover, some recent papers [23,24] point out that the Marcus model, which describes the ET dynamics in the incoherent limit [42], is not effective for a correct prediction of the ET rate constant.

Finally, it is worth noting the increasing interest in the use of silicon-based nanoparticles, functionalized with suitable organic compounds, as vectors for electrochromic functional materials. This work, where we examine the fundamental physics ruling the charge transfer in organic molecules grafted on silicon, seems well related to such a scientific research field [43,44,45,46].

## 2. Computational Details

**Figure 1:**
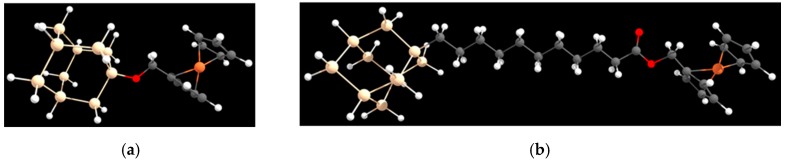
Molecular architectures grafted on the Si(111) slab: (**a**) Si_10_H_15_–O–CH_2_–FC cluster, in the text addressed as *Si_M_*–*Me–FC*; (**b**) Si_10_H_15_–(CH_2_)_10_–COO–CH_2_–FC cluster, in the text addressed as *Si_M_*–*UA*–*FC*.

DFT (Density Functional Theory) calculations were performed by using the NWChem [47] and Firefly (Firefly QC package [48]; the latter is based on the GAMESS (US) [49] source code) programs. The interfaces here studied are modeled as a silicon cluster of 10 Si atoms covalently bound to the ferrocene group by –O–CH_2_– and –(CH_2_)_10_–COO–CH_2_– alkyl chains, in the following indicated as *Me* and *UA*, respectively (see Figure 1). The valence of the silicon atoms not bound to the redox moiety is saturated with the suitable number of hydrogens. In the following, the Si_10_H_15_ moiety will be indicated as *Si_M_*. Screening full optimization geometry calculations were performed at the B3LYP/3-21G and PBE0/3-21G level of the theory, and final geometries are obtained at the PBE0/6-31G* level. Geometries optimized in the gas phase were used to perform solvation energy calculation of the various species involved in the determination of the redox potential, as proposed by Cramer and Truhlar [50,51,52]. The solute-solvent interaction was taken into account using Barone and Cossi’s polarizable conductor model (CPCM) [53]. Ionization potentials and solvation energies, needed to reckon the oxidation standard potentials, were obtained at the PBE0/6-31G* level of the theory, which can be considered a reliable level of the theory for the calculation of standard potentials [27,50,51,54,55]. Note that PBE0/6-31G* level calculations proved to produce results comparable, semi-quantitatively, to MP4/6-31G* data, when dealing with the redox reactivity of the Fe acetylacetonate complex [56]. The overall computational procedure was finalized by obtaining the data needed for the computation of the oxidation potentials, as well as the parameters required in the calculation of the physical quantities used in the Marcus theory, Equation (1). Optimization and energy values relevant to species bearing a localized charge (in particular *Fe*^1+^ and $Si_{M}^{1+}$) were calculated by using the Constrained Density Functional Theory (CDFT) method, developed by Van Voorhis and implemented in the NWChem suite of the program [32,33,47].

## 3. Charge Transfer Dynamics

The electron transfer rate constant, $k_{ET}$, is calculated using Marcus theory [23,25,47]:

(1) $$k_{ET}=\frac{2\pi }{\hbar }V_{RP}^{2}\frac{1}{\sqrt{4\pi \lambda k_{B}T}}exp\left(-\frac{\Delta E_{f}^{act}}{k_{B}T}\right)$$

The calculation of $k_{ET}$ requires knowledge of the reorganization energy (λ), the activation energy ($\Delta E_{f}^{act}$), and the electronic coupling constant, $V_{RP}$, often addressed as the “matrix element” or “direct transfer integral” [57]. λ is the sum of $\lambda_{i}$ and $\lambda_{o}$, which are the inner and outer reorganization energies, respectively: $\lambda =\lambda_{i}+\lambda_{o}$. Moreover, the following relation holds in the case of an electron transfer occurring at the electrode interface [25]:

(2) $$\lambda_{o}=\frac{e^{2}}{4\pi \varepsilon_{0}}\left(\frac{1}{a_{0}}-\frac{1}{R}\right)\left(\frac{1}{\varepsilon_{op}}-\frac{1}{\varepsilon_{s}}\right)$$

In Equation (2), $\lambda_{o}$ is computed, assuming a dielectric continuum solvent, $a_{0}$ is the reactant radius and *R* is taken as twice the distance of the centre of the molecule from the electrode surface (page 121, chapter 3.6.1) [25]. $\varepsilon_{op}$ and $\varepsilon_{s}$ are the solvent optical and static dielectric constants, respectively (in the case of acetonitrile: $\varepsilon_{op}$ = 1.8066 and $\varepsilon_{s}$ = 37.5) [58]. Moreover:

(3) $$\Delta E_{f}^{act}=\frac{\lambda }{4}{\left(1+\frac{\Delta E_{react}}{\lambda }\right)}^{2}$$

where: $\Delta E_{react}$ is the difference in energy between the reagents and the products, i.e., the thermodynamic “driving force”.

## 4. Results and Discussion

### 4.1. Standard Oxidation Potential Calculation

**Scheme 2:**
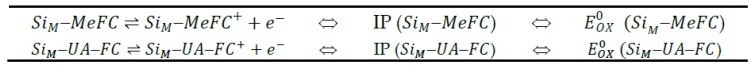
Redox reactions and relevant associated physical quantities: ionization potential (IP) and standard redox potential ($E_{OX}^{0}$).

The electrochemical behavior of the chemi-adsorbed ferrocene is assumed as a reversible single-step single-electron oxidation process, Scheme 2, where IP is the ionization potential and $E_{OX}^{0}$ is the standard oxidation potential.

**Scheme 3:**
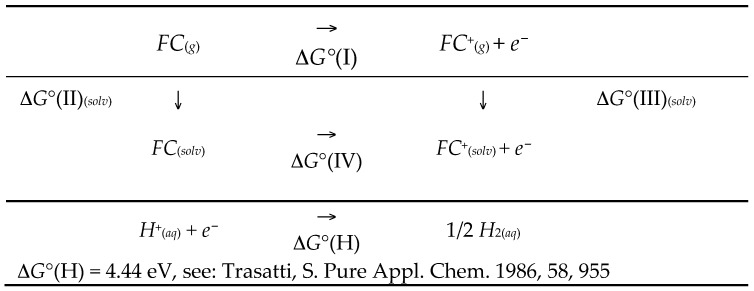
Thermodynamic cycle for the calculation of the redox potential.

The calculation of the oxidation potential is carried out by using the Nernst equation, Δ*G*° = −nFE. Then, the Gibbs standard energy variation is calculated as the sum of the individual contributions found in Scheme 3 [50]:

*H^+^*_(*aq*)_ + *FC*_(*solv*)_*⇋* 1/2 *H*_2(*aq*)_ + *FC^+^*_(*solv*)_ (4)

where FC and $FC^{+}$ stand for grafted and bulk ferrocene, respectively.

The standard Gibbs energy variation of the whole redox process, Equation (4), is determined as the sum of contributions due to the oxidation of the *FC*/*FC^+^* redox couple and hydrogen reduction, Scheme 3: Δ*G*°*_ox_*(*FC*/*FC^+^* vs. NHE) = Δ*G*°(IV) + Δ*G*°(H). In Scheme 3, step IV is the half-reaction Gibbs energy variation of the oxidation process: Δ*G*°(IV) = Δ*G*°(I) + ∆*G*°(III)_(*solv*)_ − ∆*G*°(II)_(*solv*)_. In this equation, Δ*G*°(I) is assumed to be equal to the ionization potential (IP) (see Scheme 2). This approximation was thoroughly discussed and justified in the literature [27,50,51,54,55,59]. ∆*G*°(II)_(*solv*)_ and ∆*G*°(III)_(*solv*)_ are the solvation Gibbs energies of the reduced and oxidized species, respectively (see Scheme 3). Indeed, the solvation terms in Scheme 3 refer to a bulk solvation process; however, in the present case the situation is somehow different in that the redox couple is adsorbed on the electrode surface. We calculated the solvation energy of the clusters as they are shown in Figure 1, i.e., the whole cluster is considered solvated. The solvation energy contribution of the pure silicon cluster (The part which is not in contact with the solution, i.e., the hydrogens bound to the silicon atoms) is assumed to be both negligible in absolute value, and almost constant. This term cancels out in the calculation of the whole variation of the solvation energy contribution (in the difference between the solvation energies of the reduced and oxide species, the ∆*G*°(III)_(*solv*)_ − ∆*G*°(II)_(*solv*)_ term). Gas phase optimized geometries were used for all the calculations, following the approach proposed by Cramer and Truhlar [50,51,52]. Table 1 sets out the actual data needed for the calculation of the theoretical standard potential values. $E_{OX}^{0}$ values of 0.609 V and 0.627 V vs. saturated calomel electrode (SCE) are obtained for the $Si_{M}–Me–FC$ and $Si_{M}–UA–FC$ systems, respectively. For the sake of comparison, we calculated the oxidation potential of bulk ferrocene to be $E_{OX}^{0}$ = 0.528 V vs. SCE, which has to be compared with the experimental value 0.551 V vs. SCE [60]. The good agreement between experimental and theoretical data (usually larger errors are found in the literature [27,50,54,55]) indicates that the selected level of the theory is able to “describe” in a reliable way the electronic characteristics of the species involved in our system. (We selected a 4.44 V value for the absolute hydrogen electrode potential, from Trasatti [61], which is different from the 4.42 V estimation by Tissandier [62]. See also Tripkovic and Rossmeisl for a detailed discussion on this topic [63]. Note that the experimental values are affected in general by a ±5 mV error in accuracy. Thus, it is not physically sensible to pursue a difference between experimental and theoretical potentials that is less than 70 mV.)

### 4.2. Dynamics

**Figure 2:**
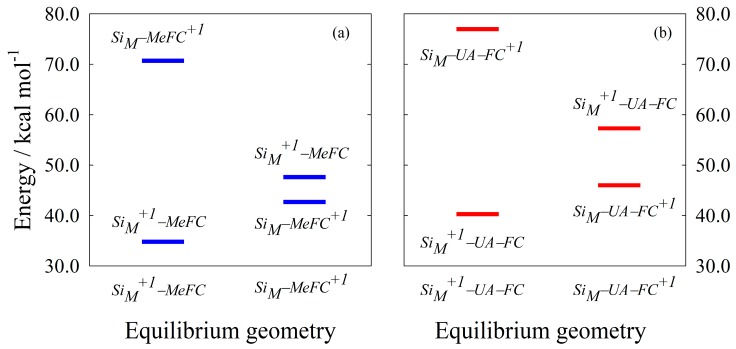
CDFT energies as a function of the initial and final equilibrium geometries. (**a**) *Si_M_–Me–FC* redox system (**b**) *Si_M_–UA–FC* redox system. Energies are scaled with respect to the relevant neutral closed shell species. See the text for details.

The Constrained Density Functional Theory (CDFT) formalism, by Wu and Van Voorhis [32,33], is exploited to build the initial and final diabatic states (see Scheme 1). The initial state is characterized by a single positive net charge constrained on the Si_10_H_15_ moiety: (*Si_M_*^1+^*–FC*). The final state is characterized by a single positive net charge constrained on the iron of the ferrocene moiety (*Si_M_–Fe*^1*+*^). In the following, the notation E(a|b) is used to indicate the energy of state a calculated at the equilibrium geometry of state b; the a and b states may or may not be the same. In any case, the equilibrium structures of both the initial and final states mentioned above were obtained with two independent CDFT geometry optimizations. Moreover, for any fixed nuclear structure, CDFT provides a direct way to calculate the energies of states with any arbitrary charge distribution [33]. The driving force ($\Delta E_{react}$) and inner-sphere reorganization energy ($\lambda_{i}$) are calculated using the CDFT paradigm according to the following relations:

(5) $$\Delta E_{react}=E\left(Si_{M}–Fe^{+}|Si_{M}–Fe^{+}\right)-E\left(Si_{M}^{+}–Fe|Si_{M}^{+}–Fe\right)$$

(6) $$\lambda_{i}=E\left(Si_{M}–Fe^{+}|Si_{M}^{+}–Fe\right)-E\left(Si_{M}^{+}–Fe|Si_{M}^{+}–Fe\right)$$

where $Fe^{+}$ indicates a +1 net charge localized on the iron, and $Si_{M}^{+}$ indicates a +1 net charge localized on the Si_10_H_15_ moiety. Figure 2 shows the energy levels of the states relevant to the Marcus two parabolas model; again it must be noted that only by using the CDFT method it is possible to calculate the energy with an arbitrary localized charge.

**Figure 3:**
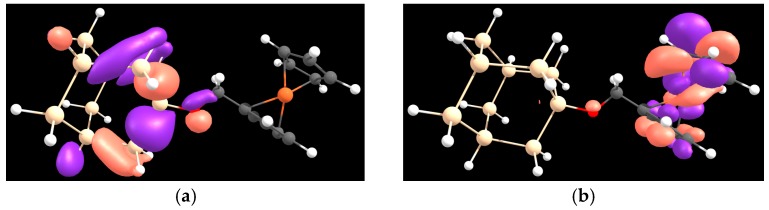
Plot of MOs involved in the $V_{RP}$ calculation for the *Si_M_–Me–FC* species. (**a**) *Si_M_*^1*+*^*–Me–FC*, molecular orbital representative of the positive charge localized on the donor state; (**b**) *Si_M_–Me–FC*^1+^, molecular orbital representative of the positive charge localized on the acceptor state.

In the present work $V_{RP}$ has been calculated at the Hartree-Fock (HF) level, exploiting the Corresponding Orbital Transformation Method (proposed by Farazdel [57]) as it is implemented in the NWChem program [47]. Figure 3 shows the relevant molecular orbitals (MOs) involved in the calculation, and the results are summarized in Table 2. At the 6-31G* level of theory, $k_{ET}=77.8\;s^{-1}$ and $k_{ET}=1.3\times 10^{-9}\;s^{-1}$, for the *Si_M_–Me–FC* and *Si_M_–UA–FC* species, respectively (see Table 2 for the values used in the calculations).

Comparison with the experimental results of similar molecular architectures shows an encouraging agreement concerning the *Si_M_–Me–FC* system (experimentally, $k_{ET}$ values are found in the 4 $s^{-1}$ to 160 $s^{-1}$ range [34,35,36,39]). On the contrary, for the *Si_M_–UA–FC* system, large differences are evident ($k_{ET}$ experimental values are found in the 30 $s^{-1}$ to 50 $s^{-1}$ range [38,39]), suggesting that some physics underlying the ET process is missing in the Marcus model. Indeed, the experimental results indicate a much faster charge transfer process than expected on the basis of the results calculated using the Marcus theory. The latter result is in line with other findings recently reported in the literature, where unexpectedly high currents are found for systems featuring ET processes occurring on long distances, and in the case of molecular spacers not “suitable” for an effective charge transport such as DNA/PNA and polypeptide systems [16,23,64,65,66].

Altogether, our theoretical findings seem to be in general agreement with results reported in the literature concerning ferrocene grafted via short alkyl chains. Indeed, charge transport through the saturated alkyl bond, just when the ferrocene is bonded via a covalent bond to the electrode, is effective enough to yield the appearance of well-defined current peaks in the experimental cyclic voltammetries (CVs) [67,68,69]. This is at variance with the case when the ferrocene redox couple is not covalently bound to the Self-Assembled Monolayer (SAM) (i.e., the ferrocene is in the bulk solution or physi-adsorbed), when CVs show current peaks which are nearly negligible [26,70]. The striking result is that the connection of the ferrocene redox couple via a single (not conjugated) covalent bond acts as an on/off switch for the ET process. To the best of our knowledge, this is one of the few works in the literature reporting theoretically calculated $k_{ET}$ values [22,40,41,71]. Remarkably, a semi-quantitative agreement between the experimental and theoretical values is found for the “short spacer” *Si_M_–Me–FC* system: our $k_{ET}=77.8\;s^{-1}$ value is comparable to experimental results obtained for quite similar molecular interfacial architectures: Dalchiele and Roth reported $150\;s^{-1}$ and $160\;s^{-1}$ $k_{ET}$ values, respectively [19,34]. An unsatisfactory agreement is instead found with the values reported by Riveros and Decker, $3.66\;s^{-1}$ and $10\;s^{-1}$ $k_{ET}$ values, respectively [35,39]. In the case of the *Si_M_–UA–FC* long spacer, the difference between theoretical and experimental values is striking. The latter result is in line with findings recently discussed in the literature, where high current values are found (CVs featuring evident redox current peaks) even dealing with long saturated alkyl-carbon chains, where a non-conducting behavior is expected [16,23,24,64,68]. Compare in particular the results discussed in the book of Nitzan [23], page 600, Figure 16.9, or Launay and Verdaguer [72], page 264.

## 5. Conclusions

In this paper, the electron transfer rate constant of two silicon-based hybrid interfaces, Si(111)/organic-spacer/Ferrocene (where the ferrocene is covalently bound to silicon via a tethering organic spacer), has been calculated exactly within the framework of the Marcus theory. In particular, the original aspect present in our results is the calculation of energies of the states featuring a net localized charge on the silicon cluster (donor state) and ferrocene moiety (acceptor state). Thus, this allows for the calculation of the energy of states featuring the same geometry, but with different charge localization, as required in the two diabatic-state parabolas of the Marcus theory (see Figure 2 and Section 4.2 for the details). The implementation of this peculiar computational strategy was possible due to the use of Constrained Density Functional (CDFT) calculations. Our theoretical results show a reasonable agreement in the case of the short alkyl spacer $k_{ET}=77.8\;s^{-1}$. Meanwhile, a striking difference with the experimental evidence is found when the long alkyl spacer is considered; $k_{ET}=1.3\times 10^{-9}\;s^{-1}$ is our calculated value. The latter result seems connected to some inadequacies present in the Marcus theory, when dealing with covalently bound donor-acceptor systems featuring a long tethering spacer. The use of long range corrected functional (accounting for states featuring prominent charge localization), or the calculation of molecular dynamics trajectories based on ab initio potential energy surfaces, are possible theoretical tools promising to yield a better agreement between theory and experiments.

## Figures and Tables

**Table 1:** PBE0/6-31G* theoretical values used to calculate the standard potential.

Redox System	Energy Ox Species, (a.u.)	∆*G*°(III)_(*solv*)_, (kcal mol^−1^)		Energy Red Species, (a.u.)	∆*G*°(II)_(*solv*)_, (kcal mol^−1^)	
		Total	Elec. ^(a)^		Total	Elec. ^(a)^
$Si_{M}–UA–FC$	−5172.168587	−5.78	−47.14	−5172.41199	35.55	−5.81
$Si_{M}–Me–FC$	−4666.305597	−5.65	−42.34	−4666.544377	33.21	−3.48
Ferrocene	−1649.745848	−40.38	−44.53	−1649.986325	1.43	−2.66

^(a)^ Electrostatic contribution.

**Table 2:** Theoretical values used in the calculation of $k_{ET}$ , Equation (1).

Redox System	$\lambda_{i}$ ^(a)^, ($\mathrm{kcal}\;mol^{-1}$)	$\lambda_{o}$ ^(b)^, ($kcal\;mol^{-1}$)	$\Delta E_{react}$ ^(a)^, ($kcal\;mol^{-1}$)	$V_{RP}$, (eV)
*Si_M_–Me–FC*	28.9	22.0	7.93	0.019
*Si_M_–UA–FC*	30.9	40.1	5.71	10^−6^

^(a)^ Calculated at the PBE0/6-31G* CDFT level of the theory; ^(b)^ calculated using Equation (2).
